# A selective LSPR biosensor for molecular-level glycated albumin detection

**DOI:** 10.1016/j.heliyon.2023.e22795

**Published:** 2023-11-28

**Authors:** Himadri Shekhar Mondal, Md Zakir Hossain, Nick Birbilis

**Affiliations:** a School of Engineering, ANU College of Engineering, Computing and Cybernetics, The Australian National University, Canberra, ACT 2601, Australia; b School of Electrical Engineering, Computing and Mathematical Sciences, Curtin University, Bentley, WA 6102, Australia; c Faculty of Science, Engineering and Built Environment, Deakin University, Waurn Ponds, VIC 3261, Australia

**Keywords:** Sensing, Glycated albumin, LSPR, Biosensor, Biosensing

## Abstract

A biosensor specifically engineered to detect glycated albumin (GA), a critical biomarker for diabetes monitoring, is presented. Unlike conventional GA monitoring methods, the biosensor herein uniquely employs localised surface plasmon resonance (LSPR) for signal transduction, leveraging a novel fabrication process where gold nanoparticles are deposited on a quartz substrate using flame spray pyrolysis. This enables the biosensor to provide mean glucose levels over a three-week period, correlating with the glycation status of diabetes patients. The sensor's DNA aptamer conjugation selectively binds GA, inducing a plasmonic wavelength shift; resulting in a detection limit of 0.1 μM, well within the human GA range of 20–240 μM. Selectivity experiments with diverse molecules and an exploration of sensor reusability were carried out with positive results. The novelty of the biosensor presented includes specificity, sensitivity and practical applicability; which is promising for enhanced diabetes diagnosis using a rapid and inexpensive process.

## 1 Introduction

Diabetes mellitus (DM) continues to result in disease-economic burden and remains a widespread public health concern. According to reports, the global prevalence of diabetes among adults was about 9.3 % in 2019, indicating that 463 million individuals were living with diabetes, with this number expected to rise to 700 million by 2045. Blood glucose, glycated albumin (GA), and glycated hemoglobin are the three main indicators used to regulate blood glucose levels in individuals with diabetes. A traditional blood glucose test determines the amount of glucose in human blood, which varies based on dietary intake, physical activity, and health status [1]. HbA1c is a blood test that estimates average glucose levels during a two-month period. Furthermore, blood glucose and HbA1c levels can vary due to medical conditions, such as thalassemia or pregnancy, rendering them less effective for diabetes diagnosis in affected individuals [2]. GA is the biomarker of choice for measuring mean glycemia, as it provides a reliable indicator of the average blood glucose level over a three-week period [3]. The activity of some hormones, such as insulin and cortisol, boosts this production. Albumin catabolism takes place near the vascular endothelium, and atrial natriuretic factor promotes its destruction. Serum albumin has several critical activities, including maintaining oncotic pressure, regulating pH, acting as an antioxidant, and transporting a variety of endogenous and exogenous substances, such as fatty acids and medicines. Serum albumin is very susceptible to post-translational changes, particularly glycation, due to its relatively lengthy half-life. Enzyme-linked immunosorbent assay (ELISA) measures are currently used in laboratory-based GA monitoring. Because of its accuracy and sensitivity, ELISA is the gold standard; yet, it is limited in terms of miniaturization, self-diagnostic application, and requires laboratory expertise [4]. In this study, glycated albumin was measured as a representative biomarker, demonstrating the miniaturized sensing platform's immediate application potential. GA has recently gained attention as a biomarker for blood glucose control in individuals with Type 1 and 2 diabetes, providing blood glucose status over approximately three weeks. The present research investigated how a glycated albumin-specific aptamer can be used to detect GA at nanomolar levels.

High-performance liquid chromatography sensors [5,[6], [7], [8]], spectrometric sensors [9,[10], [11], [12]], optoelectronic sensors [13,[14], [15], [16]], chemical analysis sensors [17,18,19], electrochemical sensors [20,21,22], and electromagnetic sensors [23] are just a few examples of the numerous different types of glucose sensors that have been reported. Electrochemical biosensors are the most commonly used type out of all the ones. However, because of surface responses, many sensors have slow response times. Because glucose has sharper peaks, less overlap, and avoids interference from luminescence and fluorescence, optical sensors, notably Raman spectra, are extremely sensitive to it [24]. However, it takes more time for this spectroscopic technique to become robust and is easily influenced by tissue layer thickness. Microwave biosensors have received a significant amount of research attention since due to their high reliability, readability, and quick reaction times [[25], [26], [27]]. Utilising electromagnetic coupling, which is a function of permittivity, glucose concentration is assessed by microwave biosensors. These biosensors have a very quick response time and can detect glucose without the use of labels. However, the sensitivity and accuracy of this technology are still constrained. Ongoing research includes the detection of molecular level conformational change. Nusz et al. [28] reported label free detection of streptavidin-biotin binding using gold nanorods. They used LSPR system for this label free detection in dark field system. They can able to detect 1 nM concentration of streptavidin which was detected by 0.59 nm mean resonant wavelength shift. Choi et al. [29] used SPR to detect dopamine (DA) using gold-silver-gold nanorods. The plasmonic shift of DA depends on the length of Au–Ag block which is decorated monoclonal antibody (Mab) against DA. Since ascorbic acid (AA) and uric acid (UA) are present in DA, they got oxidized at potential in electrochemical method and lead to false detection. Au NRs were used as a plasmonic material which includes the functionalization and blocking of the Au surface. Later DA was added and a plasmonic peak shift was recorded, the plasmonic peak was shifted from 1110 nm to 1120 nm, which indicated the binding of DA with nanorods. They used different lengths of NRs and found that for higher length of NRs there is higher binding efficiency. Gao and his research team [30] developed Surface Plasmon Resonance (SPR) based label free protein surface detection having detection limit of 0.4 pgmm^−2^. According to them, it can be commercially productive because of its transmission geometry, small footprint as well as low-cost compact spectrometer. The sensor calibration was improved by adding sensor chip and a glycerol-water solutions of different refractive indices. PBS was inserted in microfluidic channel first, later BSA solution was added and the spectrum shift was recorded, later anti-BSA solution was added and output spectrum was recorded. Ribaut et al. [31] developed a SPR biosensor for detecting Two cytokeratin 7 (CK7), which are CK7 full protein and CK7 peptide, which are important for the treatment of oncology. They achieved the detection limit for CK7 peptides is achieved as 0.4 nM. Zagorovsky and Chan [32] presented plasmonic DNAzyme strategy for pathogen detection using DNA as biomarker. They applied gold nanoparticles to detect the amplified signal because they used LSPR technology, which is straightforward for Point-of-Care Genetic Detection. In their experiment they first used Thiol modified DNA and gold nanoparticles which is needed for plasmonic spectral shift for bacteria detection using thin-layer chromatography (TLC) plate.

Cetin et al. [33] proposed a sensor which has the capacity to detect protein layer having a 3 nm thickness. According to some recent studies, it is helpful to screen a panel of biomolecular interactions, instead of a single biomolecule, for improving the accuracy of medical diagnostics. Monitoring for protein layer for diseases like Alzheimer and cancer can play a significant role for their treatment purpose. This research group develop a plasmonic chip, based on LSPR technology, which can successfully detect protein monolayer consisting of protein A/G and IgG. Plasmonic chip has microarray pixel of periodic nanoholes, which is fabricated on gold thin films, the diameter of gold nanohole is 200 nm. For designing this chip, they used 80 nm thick silicon nitride (SiN), and 120 nm thick gold layer, in between this layer, 5 nm interlayer Titanium is used for increasing efficiency of the gold layer. Lift-off free fabrication process was used for generating periodic nano holes.

Huang et al. [34] designed a biosensor which is placed in a microfluidic channel used to detect target DNA. The biosensor is based on gold nanoring and Titanium. 2 nm Titanium film and 30 nm gold film were used for designing the gold nanoring with various ion milling process for different times which helps for tuning LSPR spectral shift based on the size and shape of the gold nanoring. The spacing between nanoring was 200 nm and the inner, outer diameter of nanoring was 100 nm and 130 nm respectively. Zhang et al. [35] used LSPR for detection of streptavidin-biotin bonding. They used gold (Au) nano rods of 55 nm × 110 nm and 10 nm Titanium (Ti) nano disk. Ti is placed between the Au nanorods. The spacing between two nanorods used in that experiment was 20 nm and 45 nm, which makes them nano antenna. The difference between two nano antennas were 5 μm. Romario et al. [36] proposed Design and optimization of O^2^ microsensors to measure oxygen consumption rate (OCR) of individual pancreatic islets. Xie and co-authors [37] presented a biosensor device capable of monitoring glucose concentration having the sensitivity of 142.2 MHz/mgml^−1^.

Furthermore, colorimetry, electrochemistry, affinity chromatography, enzymatic technique, HPLC, and mass spectroscopy all have drawbacks, such as time-consuming and sophisticated analytical procedures that necessitate specialist knowledge and training, as well as the usage of chemical reagents. The spectral signals of Raman scattering are modest, whereas the scattering signal of GA is difficult to detect. As a result, using the Raman spectrum approach for qualitative and quantitative GA analysis is problematic. Like Raman, several optical methods are being used by researchers, one of them is LSPR, which used plasmonic material to generate plasmonic peak or transmission dip and later used to shift the peak to track the changed caused by molecular structural changes.

In order to induce the electromagnetic phenomenon known as localised surface plasmon resonance (LSPR), light is used to interact with metallic nanostructures like gold (Au) or silver (Ag). Nanoparticles are especially sensitive to modest local refractive index changes caused by powerful localized electromagnetic fields through LSPR [38]. The changes can be spectral shifts of extinction or scattering spectra. The commonly used nanoparticle is Au or Ag, in which Au is recommended for biological applied areas because of its inanimate characteristics, bio compatibility and thiol-gold immobilization of biomolecules. Ag has strongest and sharpest bands among all metals, but it oxidizes comparing to Au, which is another reason of not using Ag as preferred nanomaterial. The LSPR phenomena is based on the interaction of light with conductive nanoparticles are smaller comparing with wavelength, then there is an injunction between incident light and surface electrons in conduction band which generates localized plasmon oscillations with respect to resonant frequency depending on the nanoparticle size, composition and geometry [39].

Very recently LSPR based biosensors are being used as sensing platform in chemical, biochemical as well as biomedical applications because of their high performance, label-free and real-time detection of molecules by observing binding events on metal nanoparticles surface [[40], [41], [42], [43]]. For example, micro-screen printed electrodes (μSPE) for detection of GA using electrochemical techniques have been explored, which employed a bi-metallic gold-platinum (Au–Pt) nanomaterial [44]. Another study used electrochemical sensors for detection of GA using a Screen-Printed Electrode (SPE) [45]. Toshiya et al. introduced an aptamer-based capacitive electrode for electrochemical capacitance spectroscopy (ECS) to detect glycated albumin (GA) without enzymes or antibodies. Utilising a polyaryl film and poly (ethylene glycol) coating on a gold electrode, the system produces significant signals of GA against interfering proteins, successfully detecting GA concentrations of 0.1, 1, and 10 mg/mL, demonstrating its utility as a platform for enzyme-/antibody-free protein analysis [46]. Additionally, Gerard et al. [47] proposed a point-of-care (POC) system for monitoring gestational diabetes mellitus (GDM) by combining lateral flow assays (LFAs) with a handheld colorimetric reader, targeting glycated albumin (GA) as an intermediate interval biomarker. The system presents two aptamer-based assays, one for GA with a dynamic range of 3–20 mg/ml, another for serum albumin with a range of 20–50 ml/ml, and successfully integrates both into a dual assay cartridge and hence demonstrating potential as an effective platform for tracking GDM at the POC. Considering the techniques and approaches reported in what are recent literature reports, for supporting medical diagnosis in remote and low resource areas, there is a need for small, highly sensitive, and portable biosensors that use small amount of reagent and have few processing steps.

In the study herein, the development of an LSPR-based biosensor for the detection of glycated albumin (GA) is presented, revealing several new and unique features that collectively contribute to novelty. Specifically, the use of localised surface plasmon resonance (LSPR) in conjunction with a unique fabrication method involving flame spray pyrolysis is unique to the biosensor in this study. By depositing gold nanoparticles on a quartz substrate through flame spray pyrolysis, a distinctive sensing platform was created, enabling enhanced specificity sensitivity. This approach allows for a detection limit of 0.1 μM, which is within the typical human GA range, and making it suitable for practical applications in diabetes monitoring (whilst offering a quick, reliable and cost-effective procedure). Moreover, the conjugation of a DNA aptamer that selectively binds GA further emphasises the precision of the method, ensuring targeted detection. Selectivity experiments with various molecules also demonstrate the biosensor's ability to distinguish between similar compounds, while the exploration of sensor reusability underscores its practicality for repeated use. The integration of these innovative aspects represents a leap forward for biosensor, offering a more accurate, reliable, and efficient means of monitoring glycated albumin levels, a critical factor in diabetes management. coupling a novel fabrication process with state-of-the-art LSPR technology and thorough experimental validation, this work opens new avenues for enhancing biomedical diagnostics, paving the way for future advancements in personalized medicine and healthcare.

## 2 Material and methods

### 2.1 Aptamer structure and modification

The GA interacting aptamer was sourced from Biosearch Technologies (Petaluma, CA). This.

23 base pair aptamer was modified at 5′ and 3’ with amine group and Thiol group respectively (5′Amino.

C6/TGCGGTTGTAGTACTCGTGGCCG/Thiol C6 SS 3′). A stock solution of 100 μM was made with this aptamer by dissolving in Tris Ethylenediamine Tetraethyl Acetate buffer.

### 2.2 Material synthesis

At first a gold Nano-Island (AuNI) was designed using flame spray pyrolysis (FSP) setup (Fig. 2) [48]. A quartz substrate of 0.9 mm thickness was used because of its purity and high transparency in wavelength range of 400 nm–800 nm which is generally considered as visible range. Gold solution of 0.01 M was prepared by dissolving gold salt HAuCl_4_ in ethanol. The Au precursor was then fed to FSP system with a constant flow rate of 5 mL/min and the resulting in the deposition of AuNI on quartz substrate placed at 6.5 cm of height above burner (HAB).

### 2.3 Characterisation

Optical equipment and scanning electron microscopy (SEM) with a Zeiss Ultraplus (FESEM) at 3 kV were used to perform the morphological characterisation. Sensing was detected by a UV lamp, structural lenses and a light detector. Through a Pnuwave pump liquid was flown on an onto a rotating valve sensor platform (microfluidic ChipShop, Germany). Pnuwave pump was used to regulate and supply the liquid sample flow to the sensor platform.

Several responsive plasmonic metasurfaces made of restricted shape, dispersion, and mass density AuNIs were generated through a one-step process by varying the accumulation times for the Au aerosol frame from 2 to 30 s (Fig. 2). Fig. 2 illustrates the morphological classification of the resulting monolayers. While the deposition of nanoparticle aerosols has received considerable study and produced highly permeable, variational coatings of microparticles with sizes in the few nanometer range, droplets have been discovered to exhibit a very distinctive shape. The Au particles that reach the substrate fully coalesce, creating a single layer of Au nano-islands rather than three-dimensional asymmetrical structures. The latter consist, among other regular geometrical patterns, of faceted nanoprisms, nanotriangles, and nanorods. Additionally, as the deposition period is prolonged, the numerical frequency of AuNIs decreases, and their average size increases.

### 2.4 Sensing platform

**Fig. 1:**
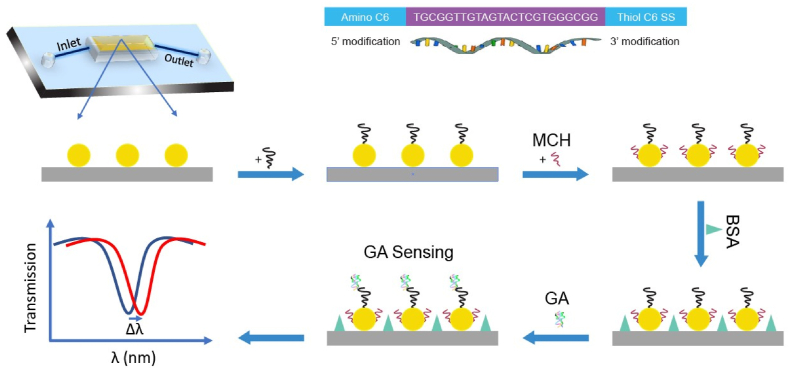
Overview of our sensing platform. At first AuNI was developed on quartz surface. Later thiol modified special sequenced DNA aptamer was deposited on gold. Quartz surface and rest of the gold surface was blocked by BSA and MCH to prevent the non-specific adsorption of surface and gold. (For interpretation of the references to color in this figure legend, the reader is referred to the Web version of this article.)

**Fig. 2:**
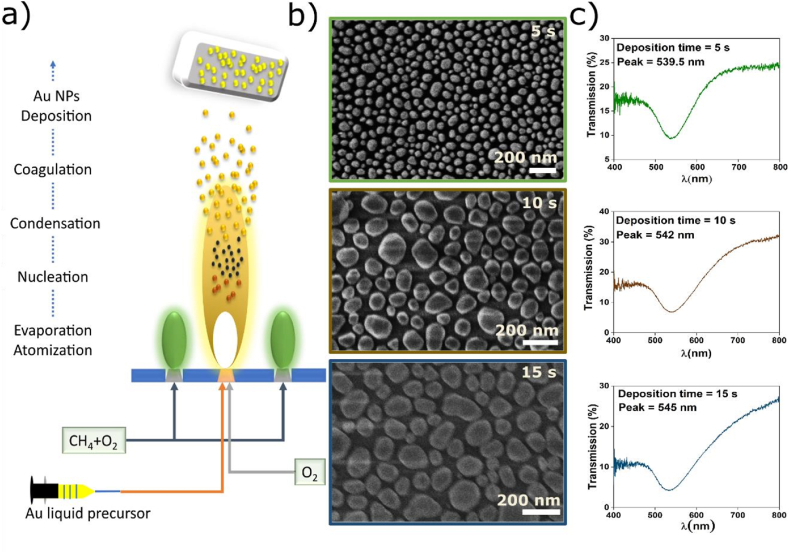
(a) Deposition of Au to form AuNI using FSP technique. FSP deposition of Au was done for different deposition time (b) SEM image of different deposition time, which indicates the size and spacing varying based on increment of deposition time. Particle size and space are much lower for lower deposition time and higher for high deposition time. (c) LSPR spectra of different Au–NI based on deposition time. It indicates lower deposition time shows better plasmonic dip as well as shape of plasmonic spectra comparing to higher deposition time.

The biosensor platform's configuration is shown in Fig. 1. It was composed of dispersed Au NIs across the surface of a quartz substrate. Au NIs are produced through a combination of aerosol deposition processes, as depicted in Fig. 2.

This scalable fabrication technique allows for the distribution of AuNIs across very large surfaces in a highly repeatable and uniform manner (organized disorder). The method is adaptable and can be used for a variety of biomarkers as target-specific aptamers can be produced [49].

The presence of glycation of human serum albumin in these areas depends on the molecular interaction with GA. The immobilization of DNA aptamers on the Au NIs causes a plasmonic peak shift. The plasmonic peak shift was shown to rise after passivating the remaining surface with a sequential deposition of 6-mercapto-1-hexanol (MCH). After blocking with bovine serum albumin (BSA), a change in peak shift was observed, indicating that the MCH/BSA passivation approach was effectively blocking the Au surfaces. For sensing in our system, we optimized the DNA aptamer concentration, and our results show that 5 μM of DNA performs as well as a higher concentration of DNA aptamer. Additionally, we used the same concentration of BSA and MCH for blocking the sensing surface and gold surface, respectively, apart from the gold-thiol bond.

Finally, GA sensing was performed by observing the plasmonic shift. The sensing platform was used for selectivity and reusability testing. Selectivity was evaluated for common interfering materials in blood, such as albumin, glucose, ampicillin, and glycine, in addition to GA. For reusability testing, the used sensing platform was heated to 400° Celsius and gently cleaned with purified water. Subsequently, sensing steps were followed, and a plasmonic shift was observed with the used sensor platform.

### 2.5 Data acquisition and analysis

The plasmonic phenomenon was observed during the continuous flow of different concentration of GA on top of DNA aptamer of Au deposited quartz substrate. During the flow of GA, continuous monitoring of data were captured every 5 s, when the plasmonic shift was in saturated position capturing data was stopped. MATLAB was used to analyze the plasmonic shift of different concentration of GA.

Data were analyzed across various GA concentrations and plotted accordingly. The human range of GA lies between 20 μM and 240 μM. our experiment started with 0.1 μM and continued till 10 μM. These experimented results indicated that after 1 μM the plasmonic shift is almost same as well as response time. Response time was also calculated for every concentration which shows that it remains almost same for last three concentration of GA. For lower concentration it takes higher response time for binding and for higher concentration it takes less time for binding of GA and DNA aptamer.

## 3 Results and discussion

**Fig. 3:**
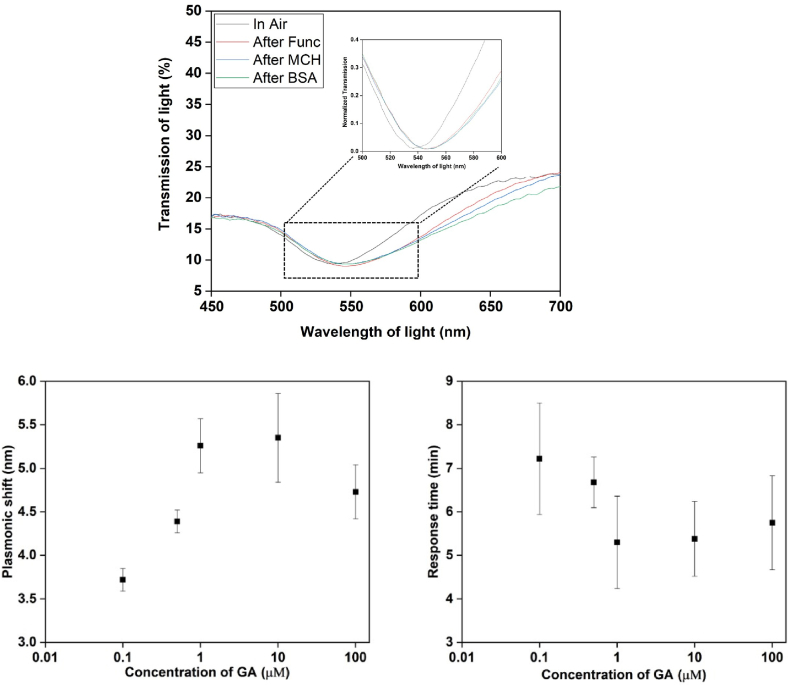
(Upper) Plasmonic spectra of functionalization and blocking stage. Each stage indicates the shift of plasmonic shift after adding a molecular level layer on top of Au. The first spectra is taken as the bare Au, which was 532 nm position. After deposition of DNA aptamer on it a big shift of 20 nm was observed. Later for blocking with MCH and BSA 1 nm shift was observed in every case. (lower left) Sensing result and (lower right) response time are presented, which indicates, after 1 μM there is a stability of plasmonic shift and the response time.

The plasmonic red shift was consistently observed at each step of the functionalization process, as depicted in Fig. 3. Initially, the pristine Au plasmonic spectrum was captured in its dry state, revealing a characteristic dip in the plasmonic spectrum at the wavelength position of 539 nm. Subsequently, as each functionalization step took place, the plasmonic red shift was observed, signifying alterations in the optical properties of the sensor. The most substantial red shift occurred after the DNA aptamer was functionalized on the bare Au surface, resulting in an approximately 10 nm shift in the plasmonic wavelength. This significant change indicates successful attachment of the DNA aptamer to the Au surface, facilitating specific molecular recognition and binding to the target glycated albumin (GA). Subsequent blocking stages showed more subtle plasmonic red shifts, typically ranging from 1 to 2 nm. These shifts are attributed to the successful blocking of any unbound sites on the Au surface, reducing non-specific binding and enhancing the sensor's selectivity for GA. The observed behaviour suggests the adsorption of GA molecules onto the surface of the gold nanoparticles (GNPs). When the GA solution was introduced and flowed on top of the GNPs, a plasmonic red shift was detected. This shift is a consequence of the change in the local dielectric environment caused by the specific attachment of GA molecules to the surface of the GNPs. The interaction between the GA molecules and the GNPs induces changes in the refractive index near the surface, leading to the observed shift in the plasmonic wavelength.

In addition to monitoring the plasmonic red shift, the transmission spectrum of the GNPs was also utilized to assess any LSPR shifts induced by varying protein deposition. This complementary analysis provided valuable information on the optical responses of the sensor to different protein concentrations, facilitating a comprehensive understanding of the sensor's detection capabilities. Remarkably, at lower concentrations of GA, the variation of the plasmonic shift was more pronounced, indicating the sensor's heightened sensitivity to trace amounts of GA. As the GA concentration increased, the degree of plasmonic shift variation decreased, implying a saturation effect at higher concentrations.

The stability of the method presented herein may be assessed from the finding that the plasmonic shifts reveal stability approaching concentrations of GA to 1 μM; which indicates a potential saturation point for the sensor. This phenomenon is likely linked to the saturation of binding sites on the sensor. It is important to acknowledge however, that numerous biological assays encounter comparable difficulties. Although these challenges can limit the sensor's dynamic range, they do not necessarily invalidate its usefulness, particularly if the sensor's primary objective is to identify lower concentrations of GA. This is particularly relevant to clinical scenarios. The feature of stabilising the plasmonic shift upon reaching a specific concentration threshold can offer certain advantages in particular applications. The observed ‘saturation’ phenomenon can be understood as the sensor attaining its maximum binding capability. The investigation of the binding kinetics of GA to the GNPs can provide additional insights into this phenomenon. By comprehending the principles of kinetics, it is possible to manipulate the sensor's characteristics in order to achieve a wider or narrower dynamic range, contingent upon the specific application.

Linearity is an important feature for an ideal sensor – whereby it is favourable to achieve a measured response that remain proportional and consistent throughout a broad spectrum of concentrations. However, the presence of non-linearity at elevated concentrations, could perhaps be attributed to the saturation effect previous described. The phenomenon of non-linear response, which is exhibited at greater concentrations, can be effectively addressed by maximising the density of DNA aptamers on the gold (Au) surface. An increased density has the potential to expedite saturation, whilst a decreased density has the potential to expand the linear range of the sensor. Furthermore, the incorporation of a calibration phase within the sensing technique may yield advantages by addressing non-linearities and enhancing the precision of the sensor.

The utilization of localised surface plasmon resonance (LSPR) for sensing is an established idea in scientific research; however, the particular application of LSPR for the detection of GA and the observed increased sensitivity towards minute quantities are innovative characteristics of this study. The novelty of this study is not only in the utilization of localised surface plasmon resonance (LSPR) for the detection of genetic anomalies (GA), but also in the notable increase in sensitivity obtained at lower concentrations. The considerable therapeutic value of this heightened sensitivity at low concentrations is particularly evident in early diagnosis situations, wherein the concentration of the target molecule is minimal. In addition, the employment of DNA aptamers designed for GA recognition introduces an additional level of specificity to the sensor, so differentiating it from conventional gold nanoparticle-based localised surface plasmon resonance (LSPR) sensors.

**Fig. 4:**
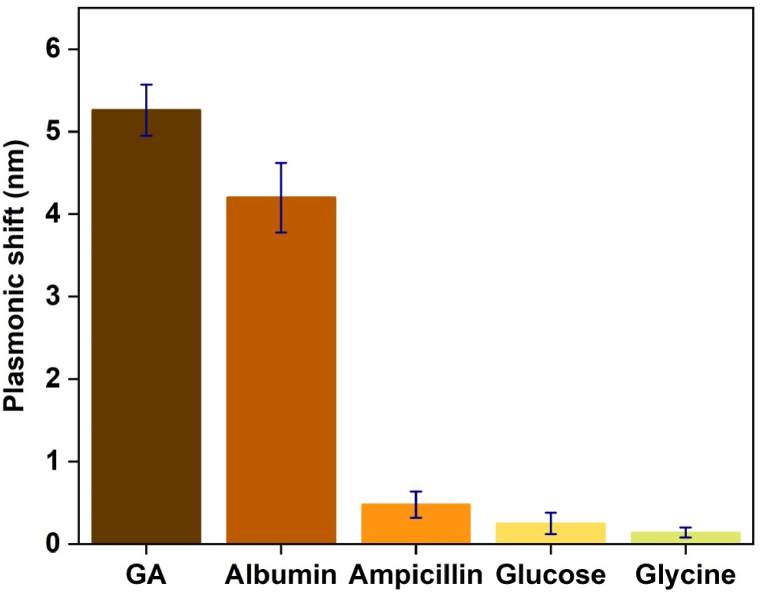
Selectivity test of common interfering molecules found in blood. Concentration is 1 μM for each molecule. It shows the higher plasmonic shift with GA which is close to 5.5 nm but the second one is albumin which is close to 4.5 nm. Rest three molecules shows very less response, less than 1 nm.

Selectivity testing was also performed and is presented in Fig. 4. Various interfering compounds common in blood circulation, such as HSA, Ampicillin, Glucose and Glycine were used to test the specificity of the optical sensor platform in contrast to GA. The result indicates that in the case of GA, the highest plasmonic shift was observed close to 5.5 nm, whilst HSA was observed close to 4.2 nm. Conversely, Ampicillin, Glucose and Glycine showed less plasmonic shift (below 1 nm).

The average LD for the sensor was determined to be 0.1 μM. Given that GA has a molecular weight of 66.5 kDa, the sensor's LD is performing well considering technological aspects. There have been reports of GA-based sensors in earlier investigations. A research group [50] was able to detect 0.47 mg/ml in an enzymatic test and 50 g/ml in a graphene-based aptasensor, which performs better in terms of limit of detection but in the consideration of quick and cost-effective detection platform this LSPR based sensor performed well [51]. For GA detection various methods were applied to achieve the LD, some of them are electrochemiluminescence sensor (0.1 μM (6.6 μg ml^−1^)) [52], Enzymatic assay sensor (0.36 mg/ml) [53], Electrochemical (3 μg ml^−1^) [54], Raman spectroscopy sensor (13.7 μM) [55], colorimetric sensor based on Hydrazine (0.7 μM) [56], immunoturbimetric sensor focused on chromatography (0.81 mg l^−1^) [57]. GA content in HS was also discovered to be 3–105.3 μM (0.2–7.0 mg/ml) [50,51]. Hence it may be stated that the sensor can be used for GA detection from diabetic serum. Furthermore, the apta-sensor is also shown to be able to detect the presence of GA in diabetic serum.

The principle of developing an LSPR based plasmonic biosensor in this research was to realise a cost effective and quick detection method. The stability of this system for lower concentrations of glycated albumin, exhibits slight variations that posed a challenge to precise determination of the limit of detection for the Glycated Albumin sensor. The sensor's ability to accurately detect and quantify lower levels of glycated albumin is affected by these fluctuations, making it somewhat difficult to establish a definitive lower limit of detection. Despite the inherent challenges in pinpointing this threshold, efforts are being made to improve the sensor's performance and establish a more robust detection range for glycated albumin at lower concentrations.

The sensor type studied herein didn't reach as low a limit of detection as different methods found in literature [58] but could detect within the human range which is a particularly significant outcome from this research; whilst this research used promising LSPR technology which was a quick and easy process. Some other methods may have lower detection limit but the practical implementation is not easy as LSPR based one. Ongoing research and calibration adjustments aim to enhance the sensitivity and reliability of the sensor, ensuring its suitability for accurate measurements in the clinical context and facilitating its potential integration into diagnostic applications for monitoring glycemic control and early detection of diabetes-related complications.

**Fig. 5:**
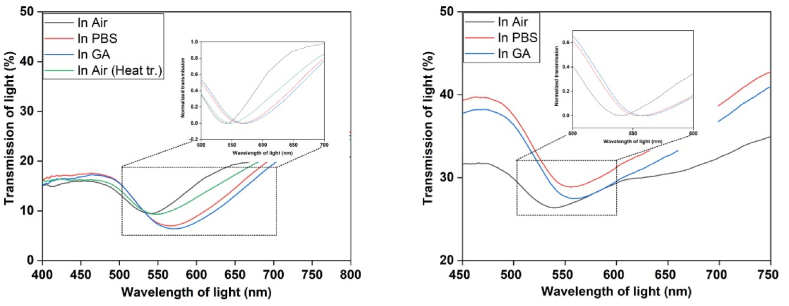
For reusing of this sensor, after heat treatment same steps were performed starting from DNA deposition and it shows after heat treatment the plasmonic shift was back to the same position almost, which indicates that all the Sensing molecules including GA and DNA aptamer was removed from the surface. Later GA detection was performed with 1 μM concentration and plasmonic shift was observed with the GA but slightly less shift comparing to the fresh sensing platform.

The reusability of the sensor type studied herein was also tested. For testing reusability, a quartz substrate was held at 400° C for 20 min and then washed with clean water. Later the same step for sensing was followed and found the sensing can still be done using this quartz substrate but a bit less plasmonic shift was observed (Fig. 5).

The observation of consistent plasmonic red shifts during each functionalization step highlights the successful modification of the sensor surface and its potential for sensitive detection of GA. The ability to detect minute changes in GA concentrations at lower levels demonstrates the sensor's promising applications in early disease diagnosis and monitoring. Gold (Au) metasurfaces can be a good platform for optical biosensor. It's been a while demonstrated that plasmonic structures can be an excellent platform. Due to the unique properties of Au nanostructures, they are ideal for biomolecular sensing. their resistance to oxidation. The plasmonic performance in liquid environments, Au sensors have been extensively researched. By leveraging the interactions between GNPs and GA, this innovative plasmonic sensor holds significant promise for future advancements in biomedical research, disease management, and personalized medicine. However, further optimszation and validation are required to realise the full potential of the presented sensor as a reliable and versatile tool for clinical diagnostics and point-of-care applications. However, achieving high sensitivity in this domain remains challenging. Because of the minimal refractive index changes associated with changes in gas molecule concentration, the gas phase is preferred, especially for non-functionalized (label-free) surfaces.

### 3.1 Limitations identified from the current study and future directions

Despite the development of the LSPR based cost-effective sensor, it is essential to acknowledge that the sensor does come with a number of drawbacks that warrant attention and improvement. One of the primary concerns lies in the instability of the production process for AuNI, which poses challenges in ensuring consistent sensor performance. The synthesis of AuNI involves intricate procedures, and the reproducibility of the nanocages' size, shape, and surface properties can be affected by subtle variations in reaction conditions. This unpredictability hampers the reliability of the sensor's response, necessitating stringent quality control measures during production and comprehensive characterization of each batch of AuNI. Additionally, the thin layer of Au in the sensor demands careful handling during the deposition of DNA aptamers. The delicate nature of the thin gold film requires precise and controlled deposition techniques to ensure uniform coverage with DNA aptamers. Any mishandling or uneven deposition could lead to irregularities in the binding sites, causing fluctuations in the sensor's sensitivity and specificity. Addressing this challenge involves refining the fabrication process to enhance the adhesion and stability of the Au layer while ensuring uniformity across the entire sensing surface.

Furthermore, the deposition time of the DNA aptamer is critical to consider. The current 4-h deposition time, while sufficient for successful binding with glycated albumin (GA), also presents a potential issue. Prolonged exposure beyond this time frame could lead to the degradation of the DNA aptamers due to environmental factors, such as temperature and humidity. Consequently, the aptamer's structural integrity and binding affinity with GA might diminish, ultimately resulting in less pronounced plasmonic shifts and decreased sensor sensitivity. Fine-tuning the deposition time and optimizing the environmental conditions are essential to maximize the aptamer's stability while ensuring robust and reliable sensor responses. Moreover, the limited number of GA concentrations tested in the current study is another noteworthy limitation. The five concentrations used might not fully represent the dynamic range of GA concentrations encountered in real-world samples. Variations in GA levels can occur in various physiological and pathological conditions, and the sensor's efficacy across this broader concentration spectrum needs to be assessed. Conducting experiments with a more extensive range of GA concentrations will enable the characterization of the sensor's dynamic response, detection limit, and quantification capabilities, providing valuable insights into its practical utility for diverse clinical scenarios.

While the LSPR based cost-effective sensor represents a promising advancement in the field, its current state demands addressing the mentioned drawbacks to enhance its stability, sensitivity, and reliability. Collaborative efforts from researchers and engineers are required to refine the sensor's production process for AuNI, optimize the deposition time of DNA aptamers, and broaden the concentration range of GA experiments. Through continuous improvements and iterations, the sensor can be transformed into a powerful and versatile tool for sensitive and precise glycated albumin detection, contributing significantly to biomedical research and clinical diagnostics. This enhanced sensor technology has the potential to play a significant role in disease monitoring, early diagnosis, and personalized treatment strategies.

## 4 Conclusion

The work herein presented a biosensor for detection of glycated albumin (GA), along with its exploitation as a biomarker for diabetes. The effects of protein binding on the Localized Surface Plasmon Resonance (LSPR) of AuNI were explored in this research. The novel biosensing device explored herein was capable of providing qualitative data on the presence of molecules in solution, and not quantitative information regarding their properties (e.g., size, weight). However, a flexible technique for designing nanomolar detection limits has been provided, along with demonstration of selective biosensors. The presented GA biosensor arrangement yields a linear response in the lower GA range, with outstanding selectivity against other typical biomolecules found in human blood, such as human serum albumin, ampicillin, glycine, and glucose. When used in conjunction with the standard approach of glucose monitoring, this sensor may also be more effective in diabetes diagnosis. In future work, several parameters may be changed (such as the DNA aptamer, silver surface, and environment of experiment) to make the sensor more effective for the detection of GA from human blood.

## Data Availability

Data will be made available on request.

## Additional information

No additional information is available for this paper.

## Funding

This research is supported by Our Health in Our Hands, A strategic initiative of 10.13039/501100000995Australian National University, Canberra, Australia.

## CRediT authorship contribution statement

**Himadri Shekhar Mondal:** Visualization, Validation, Software, Resources, Methodology, Investigation, Formal analysis, Conceptualization, Writing - original draft, Writing - review & editing. **Md Zakir Hossain:** Visualization, Validation, Supervision, Software, Resources, Project administration, Methodology, Investigation, Writing - review & editing. **Nick Birbilis:** Validation, Supervision, Software, Resources, Methodology, Investigation, Funding acquisition, Writing - review & editing.

## Declaration of competing interest

The authors declare that they have no known competing financial interests or personal relationships that could have appeared to influence the work reported in this paper.
